# Supplementary material for: A deep learning approach for complex microstructure inference
Source: Nat Commun. 2021 Nov 1;12:6272. doi: 10.1038/s41467-021-26565-5 (PMC8560760; doi:10.1038/s41467-021-26565-5)
Supplement: Supplementary file 1 — Supplementary Information [file 41467_2021_26565_MOESM1_ESM.pdf]

Supplementary Information for "A Deep Learning Approach for Complex Microstructure Inference"

by
Ali Riza Durmaz[1,2,3,*,+], Martin Müller[4,5,+] Bo Lei[6], Akhil Thomas[1,3], Dominik Britz[4,5], Elizabeth A. Holm[6], Chris Eberl[1,3], Frank Mücklich[4,5], and Peter Gumbsch[1,2]

1: Fraunhofer Institute for Mechanics of Materials IWM, Freiburg, 79108, Germany
2: Karlsruhe Institute of Technology (KIT), Institute for Applied Materials IAM, Karlsruhe, 76131, Germany
3: University of Freiburg, Freiburg, 79110, Germany
4: Saarland University, Department of Materials Science, Saarbrücken, 66123, Germany
5: Material Engineering Center Saarland, Saarbrücken, 66123, Germany
6: Carnegie Mellon University, Department of Materials Science and Engineering, Pittsburgh, PA 15213, USA

*: ali.riza.durmaz@iwm.fraunhofer.de
+: these authors contributed equally to this work

Supplementary Table 1: Number of images and their lath-shaped bainite content for each fold of each of the four data sets.

Dataset	Fold	0	1	2	3	4
LOM	Number of tile images	151	151	151	151	150
	Bainite pixel percentage [%]	27	26.3	27.4	31.2	29.8
LOM downscaled	Number of tile images	36	36	36	36	35
	Bainite pixel percentage [%]	30.6	26.7	25.2	28	25.4
SEM	Number of tile images	83	83	83	82	82
	Bainite pixel percentage [%]	50.9	58.6	62.3	63.2	60.2
SEM downscaled	Number of tile images	27	27	27	27	27
	Bainite pixel percentage [%]	50.9	46.8	55.3	55.1	59.5

Supplementary Table 2: Number of images and their lath-shaped bainite content for each fold of each of the data sets from tile experiment.

Tile size	Fold	0	1	2	3	4
64	Number of tile images	2612	2611	2611	2611	2611
	Bainite pixel percentage [%]	27.2	27.7	27.1	27.8	27.5
96	Number of tile images	1020	1020	1020	1020	1020
	Bainite pixel percentage [%]	27.0	29.7	27.7	29.3	28.1
128	Number of tile images	653	653	653	653	652
	Bainite pixel percentage [%]	27.3	27.2	27.2	28.7	26.9
192	Number of tile images	255	255	255	255	255
	Bainite pixel percentage [%]	28.4	28.5	29.0	27.3	28.7
256	Number of tile images	164	163	163	163	163
	Bainite pixel percentage [%]	25.8	28.4	27.1	28.5	27.4
512	Number of tile images	41	41	41	41	40
	Bainite pixel percentage [%]	27.6	29.0	27.4	29.1	24.1

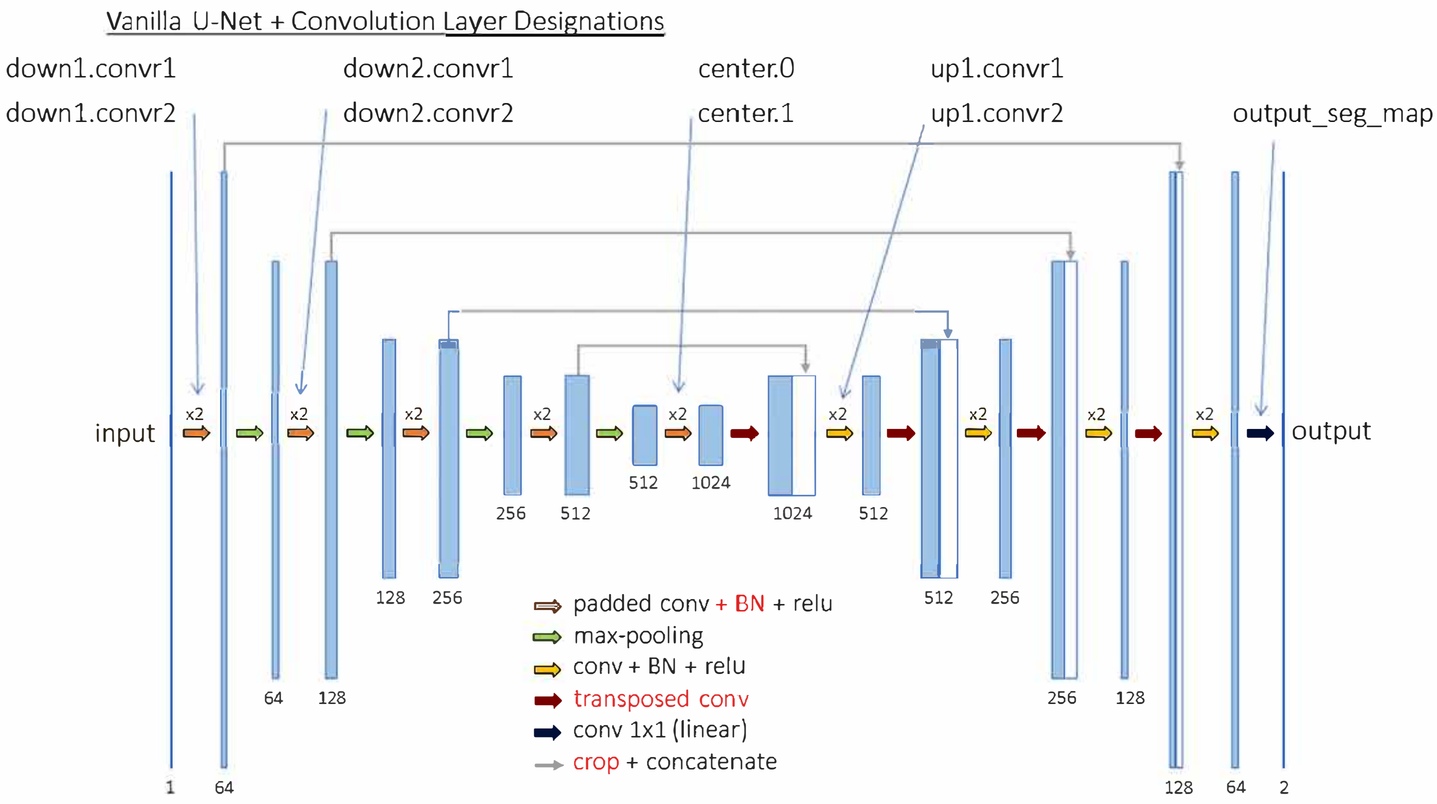

Supplementary Figure 1: Vanilla U-Net architecture.

U-Net VGG16

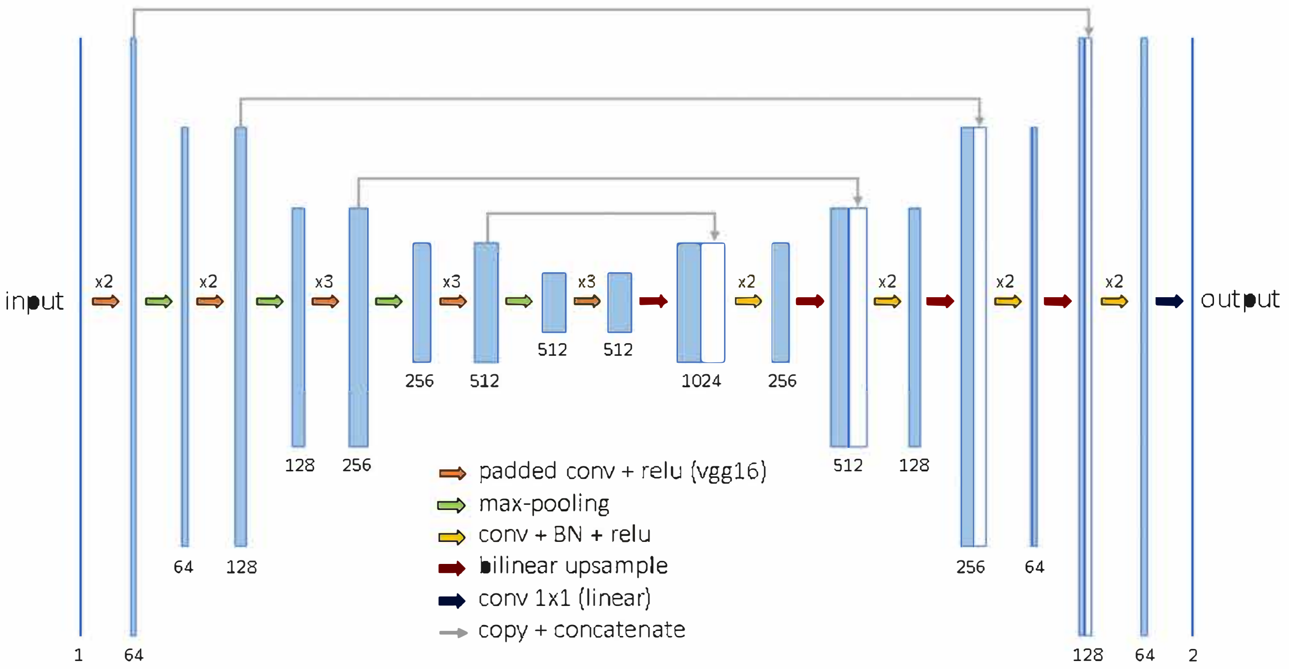

Supplementary Figure 2: VGG16 U-Net architecture.

Supplementary Table 3: Description of individual augmentations applied for vanilla U-Net models on LOM images.

Augmentation type	Description: aug. type / parameter(s)	x_{lim}	p
Affine transformation	Linear transformation / rotate, shift and scale limit	30, 0.1, 0.1	0.8
Rotation 90 °	- / -	-	0.25
Reflection	- / -	-	0.25
Elastic transformation	Local deformations / alpha affine, alpha, sigma, approx.	0, 15.86, 5.15, True	0.35
Optical distortion	Barrel or pincushion / distort limit, shift limit	0.07, 0.23	0.25

Table 2: Description of individual augmentations applied for vanilla U-Net models on SEM images

Augmentation type	Description: aug. type / parameter(s)	x_{lim}	p
Affine transformation	Linear transformation / rotate, shift and scale limit	30, 0.1, 0.1	0.8
Rotation 90 °	- / -	-	0.25
Reflection	- / -	-	0.25
Elastic transformation	Local deformations / alpha affine, alpha, sigma, approx.	0, 40.52, 7.77, True	0.35
Optical distortion	Barrel or pincushion / distort limit, shift limit	0.14, 0.42	0.25
Gaussian blurring	Convolution Gaussian kernel / blur kernel size	9	0.2
Motion blurring	Convolution motion-blur kernel / blur kernel size	7	0.2

Supplementary Table 4: Description of augmentations applied for UNet-Vgg16 models.

Augmentation type	Description: aug. type / parameter(s)	x_{lim}	p
Affine transformation	Linear transformation / rotate, shift and scale limit	45, 0, 0.2	0.7
Reflection	- / -	-	0.5
Elastic transformation	Local deformations / alpha affine, alpha, sigma, approx.	0, 40, 6, True	0.5
Grid Distortion	Local deformations / num steps, distort limit	5, 0.2	0.5
Gaussian blurring	Convolution Gaussian kernel / blur kernel size	7	0.2
Motion blurring	Convolution motion-blur kernel / blur kernel size	5	0.2
Gaussian noise	- / var limit	(10,50)	0.2
Contrast	- / limit	0.15	0.7
Brightness	- / limit	0.1	0.7

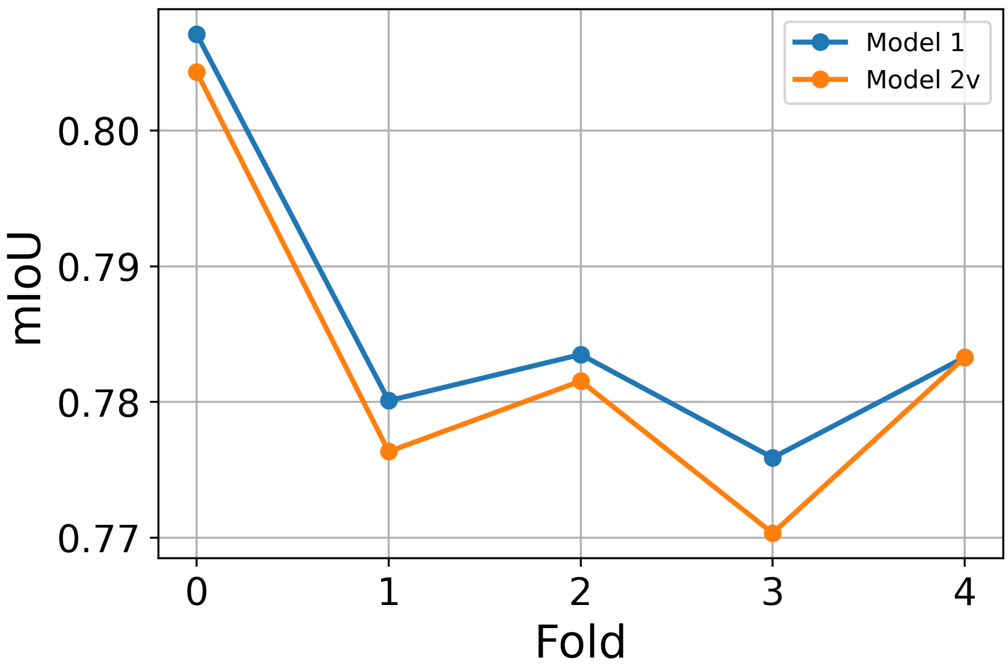

Supplementary Table 3: Class-averaged IoU for the different folds for two exemplary models.

Supplementary Table 5: Description of individual training +validation as well as testing times for both architectures in the best configurations.

	Vanilla U-Net		VGG16 U-Net	
Dataset	Train + validation	Test on full scale images	Train + validation	Test on full scale images
LOM	256×256, 8 ms/image	1024×1024, 92 ms/image	256×256, 47 ms/image	1024×1024, 240 ms/image
SEM	512×512, 134 ms/image	1024×717, 74 ms/image	512×512, 200 ms/image	1024×717, 160 ms/image

Supplementary Figure 4: Grad-CAM maps indicating image regions that dictated the decision of the Vanilla U-Net network with respect to the background class. The individual panel captions refer to the specific network layer for which the activation map is computed (see Supplemental Figure 1).

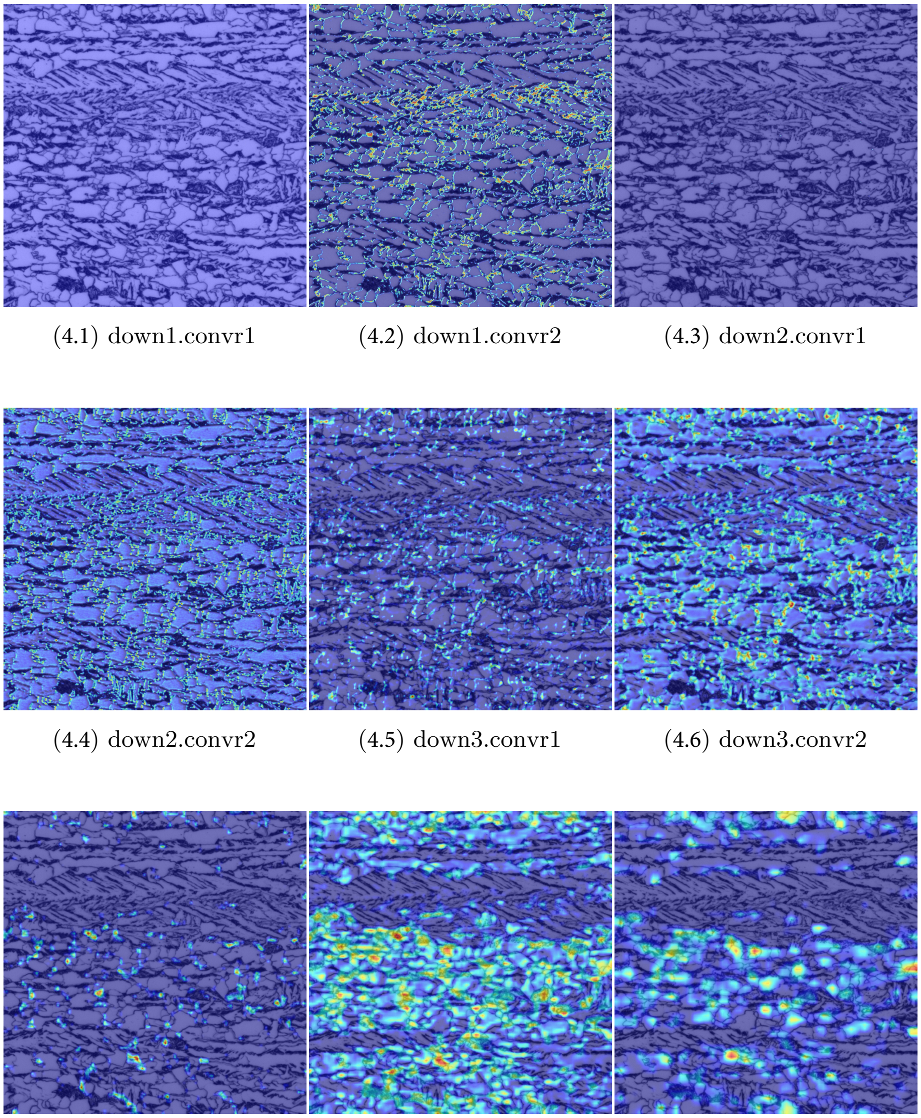

(4.1) down1.convr1 (4.2) down1.convr2 (4.3) down2.convr1

(4.4) down2.convr2 (4.5) down3.convr1 (4.6) down3.convr2

(4.7) down4.convr1 (4.8) down4.convr2 (4.9) center.0

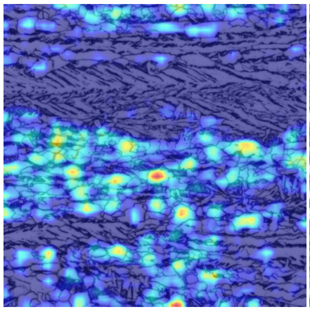
(4.10) center.1

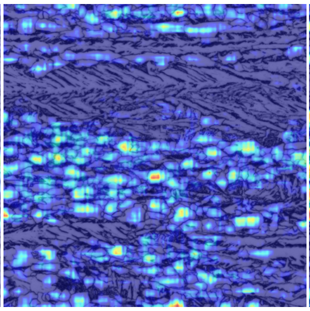
(4.11) up1.convr1

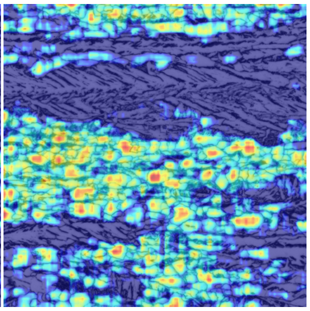
(4.12) up1.convr2

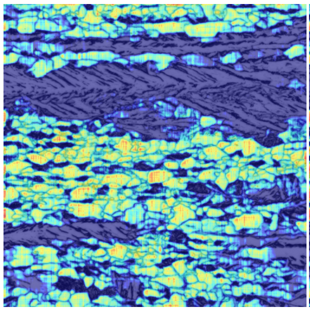
(4.13) up2.convr1

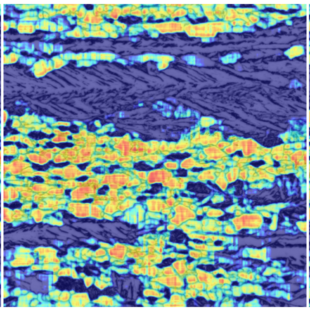
(4.14) up2.convr2

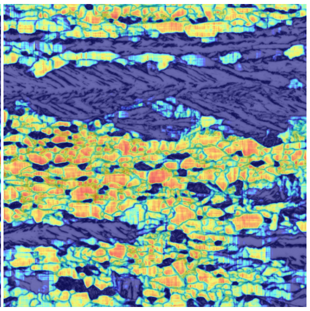
(4.15) up3.convr1

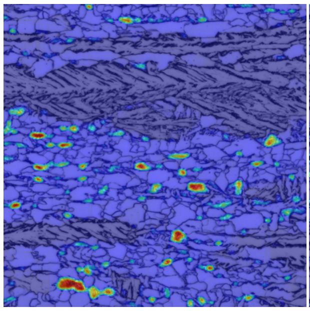
(4.16) up3.convr2

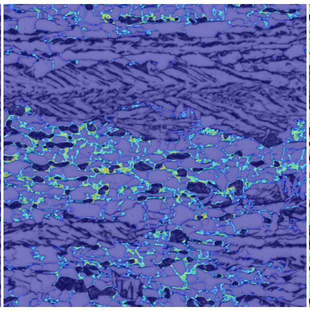
(4.17) up4.convr1

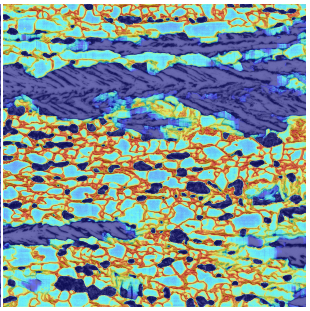
(4.18) up4.convr2

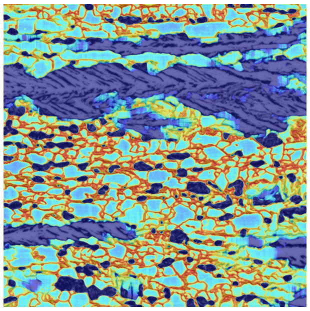

(4.19) output.seg.mask

Supplementary Figure 5: Grad-CAM maps indicating image regions that dictated the decision of the Vanilla U-Net network with respect to the foreground class. The individual panel captions refer to the specific network layer for which the activation map is computed (see Supplemental Figure 1).

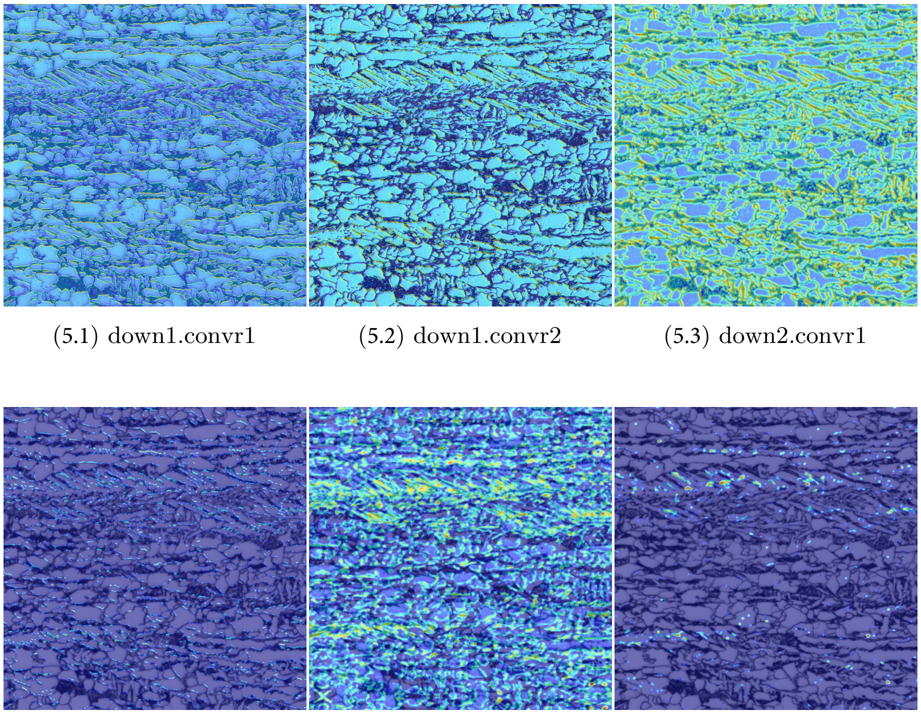

(5.1) down1.convr1 (5.2) down1.convr2 (5.3) down2.convr1

(5.4) down2.convr2 (5.5) down3.convr1 (5.6) down3.convr2

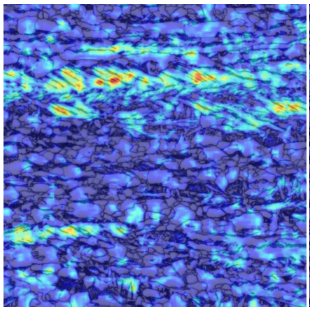
(5.7) down4.convr1

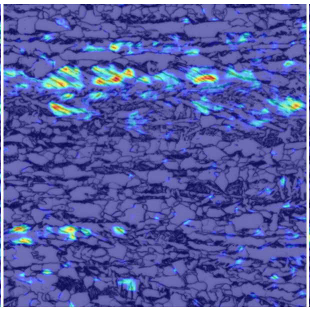
(5.8) down4.convr2

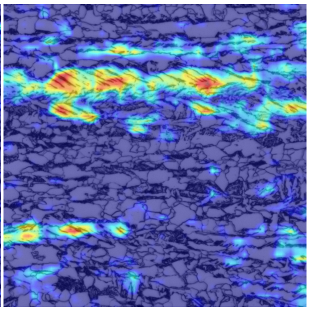
(5.9) center.0

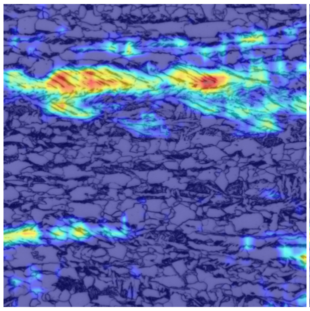
(5.10) center.1

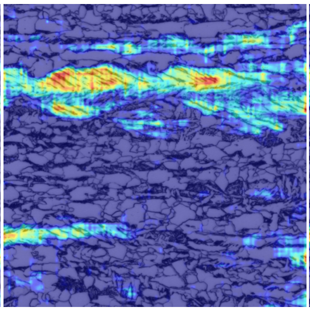
(5.11) up1.convr1

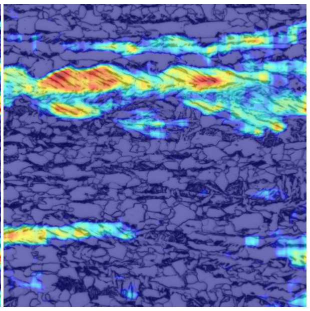
(5.12) up1.convr2

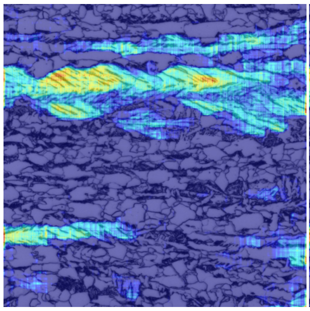
(5.13) up2.convr1

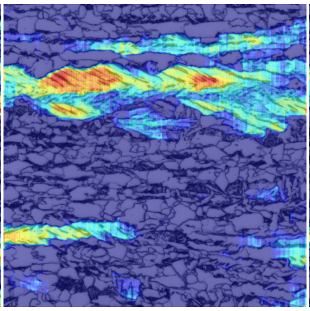
(5.14) up2.convr2

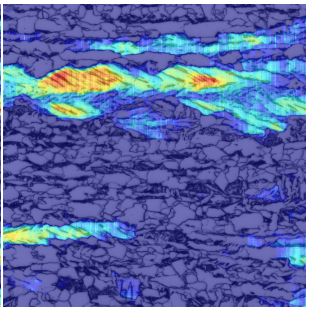
(5.15) up3.convr1

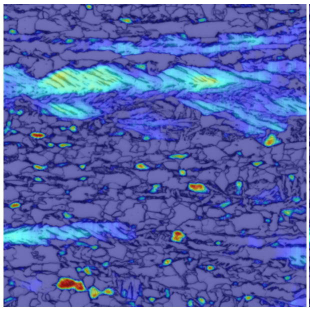

(5.16) up3.convr2

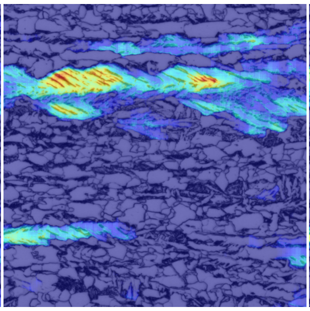

(5.17) up4.convr1

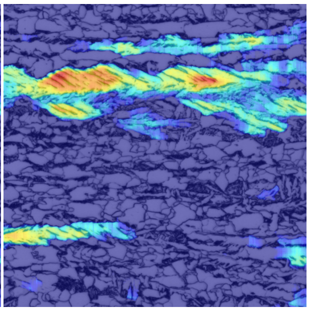

(5.18) up4.convr2

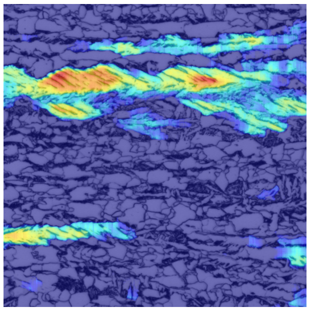

(5.19) output.seg.mask

Supplementary Figure 6: Grad-CAM maps indicating image regions that dictated the decision of the VGG U-Net network with respect to the background class. The individual panel captions refer to the specific network layer for which the activation map is computed (see Supplemental Figure 1 and 2).

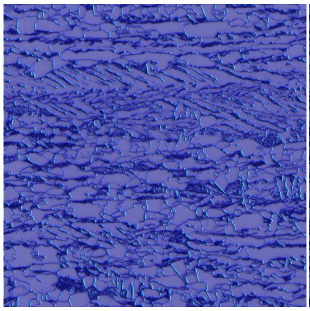

(6.1) down1.convr1

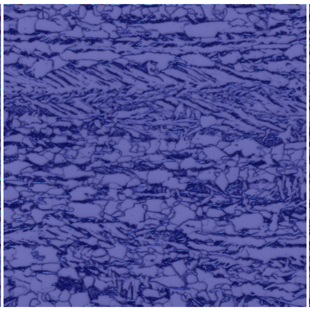

(6.2) down1.convr2

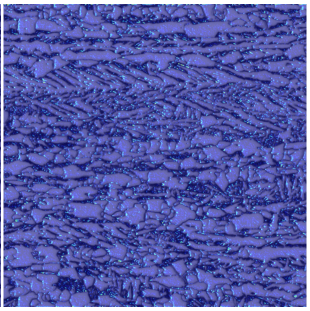

(6.3) down2.convr1

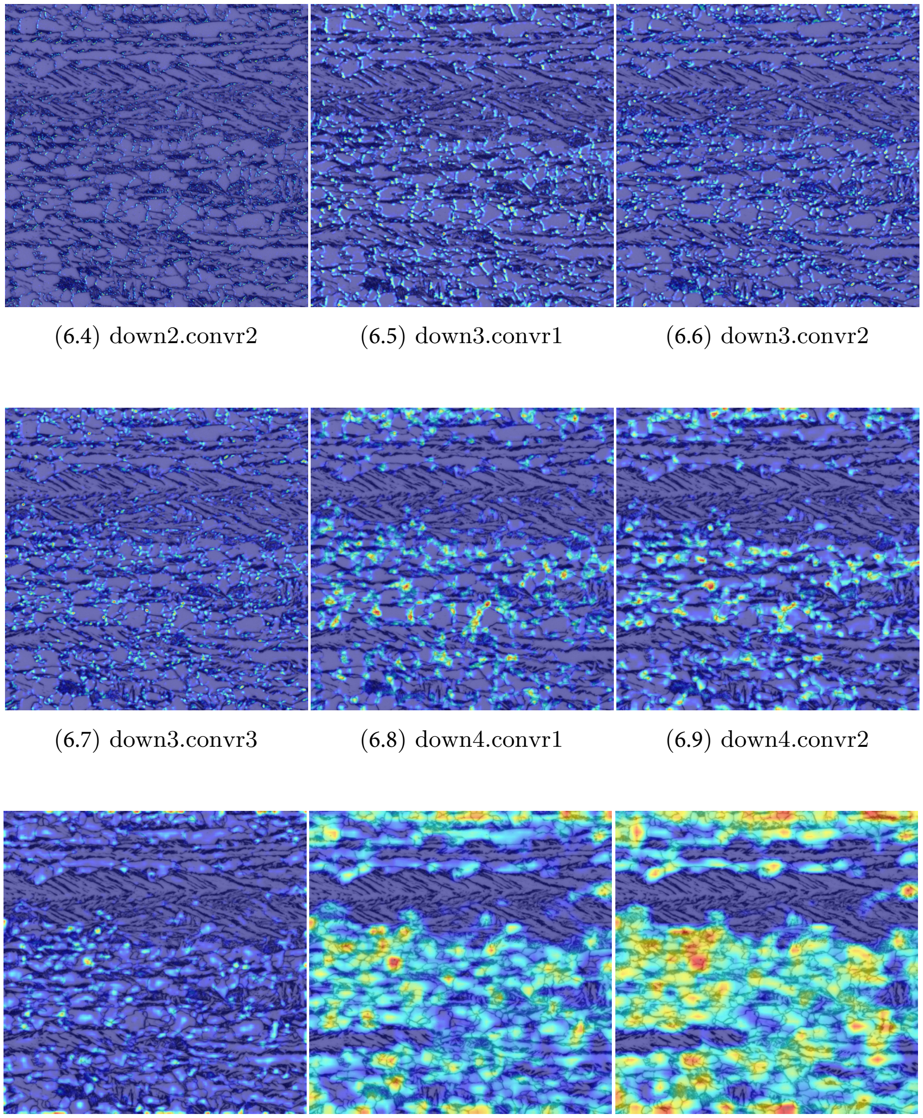

(6.4) down2.convr2 (6.5) down3.convr1 (6.6) down3.convr2

(6.7) down3.convr3 (6.8) down4.convr1 (6.9) down4.convr2

(6.10) down4.convr3 (6.11) center.0 (6.12) center.1

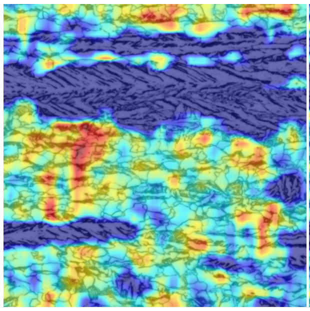
(6.13) center.2

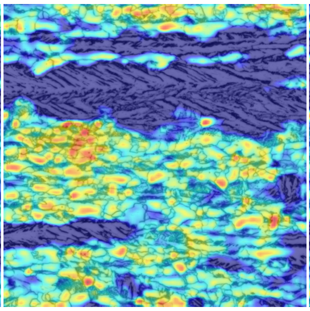
(6.14) up1.convr1

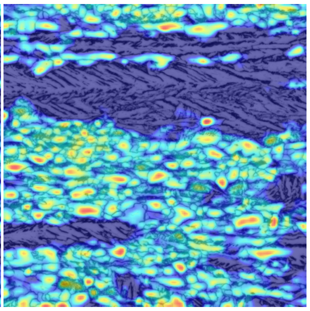
(6.15) up1.convr2

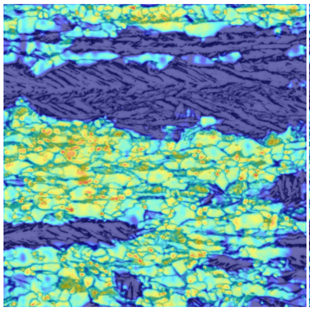
(6.16) up2.convr1

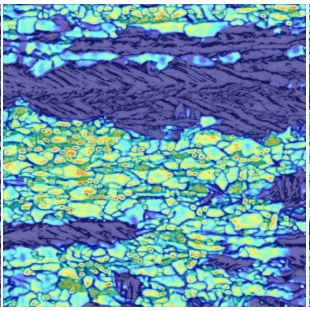
(6.17) up2.convr2

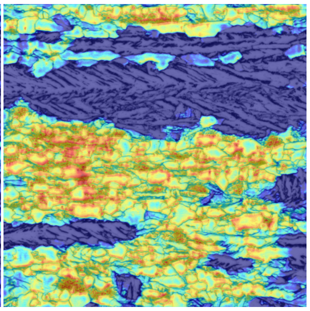
(6.18) up3.convr1

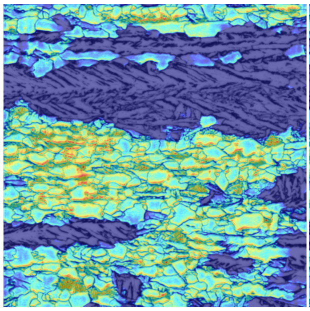
(6.19) up3.convr2

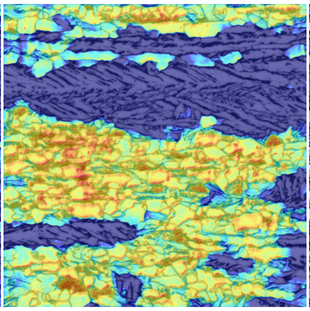
(6.20) up4.convr1

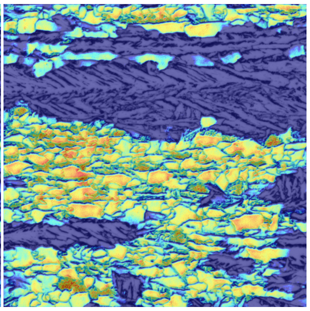
(6.21) up4.convr2

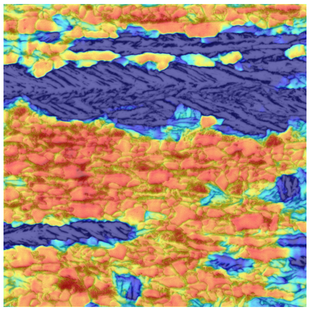

(6.22) output.seg.mask

Supplementary Figure 7: Grad-CAM maps indicating image regions that dictated the decision of the VGG U-Net network with respect to the foreground class. The individual panel captions refer to the specific network layer for which the activation map is computed (see Supplemental Figure 1 and 2).

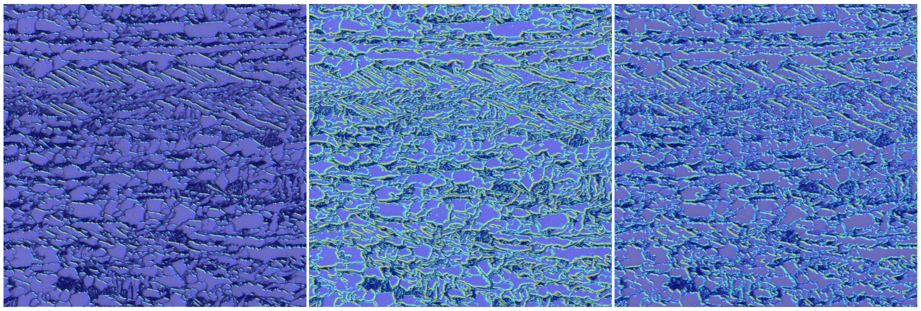

(7.1) down1.convr1 (7.2) down1.convr2 (7.3) down2.convr1

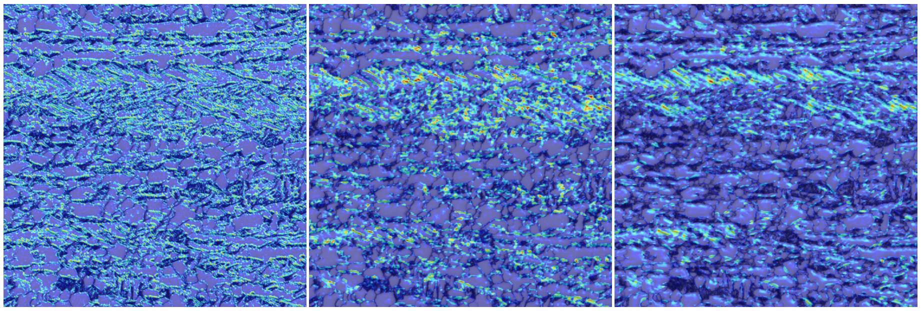

(7.4) down2.convr2 (7.5) down3.convr1 (7.6) down3.convr2

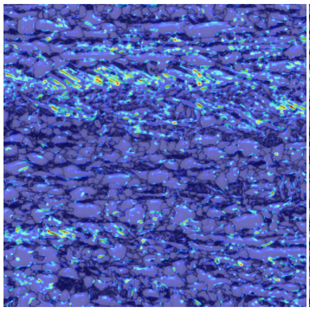

(7.7) down3.convr3

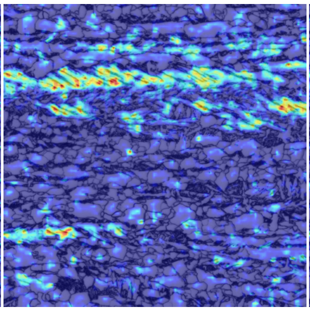

(7.8) down4.convr1

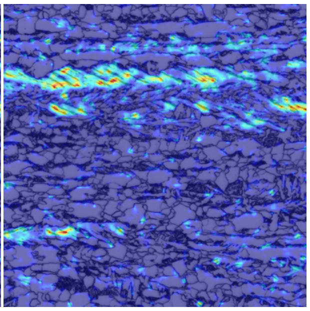

(7.9) down4.convr2

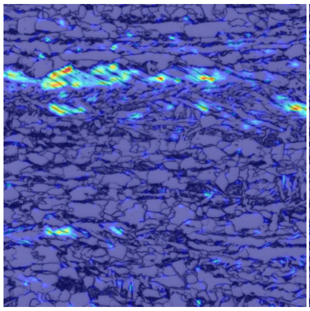

(7.10) down4.convr3

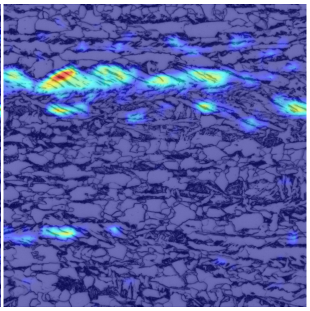

(7.11) center.0

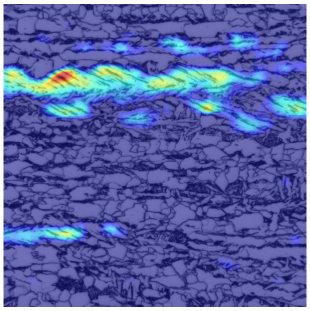

(7.12) center.1

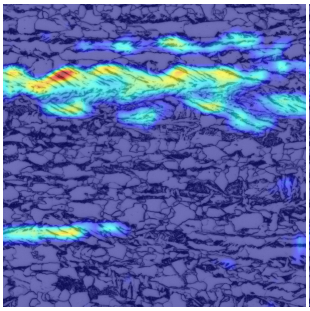

(7.13) center.2

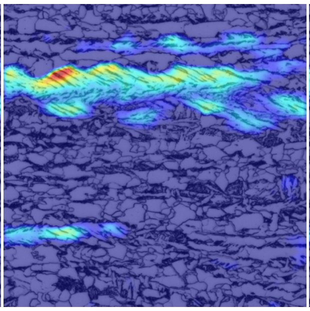

(7.14) up1.convr1

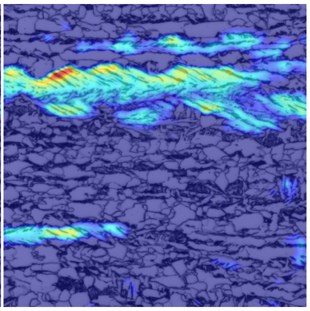

(7.15) up1.convr2

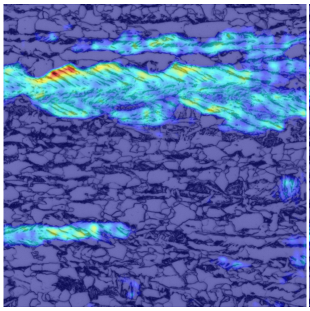

(7.16) up2.convr1

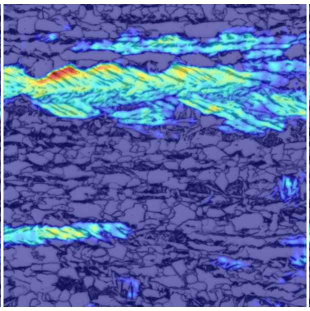

(7.17) up2.convr2

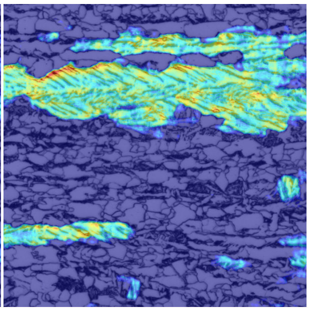

(7.18) up3.convr1

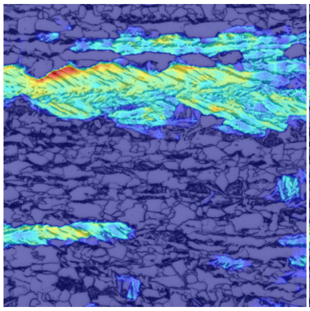

(7.19) up3.convr2

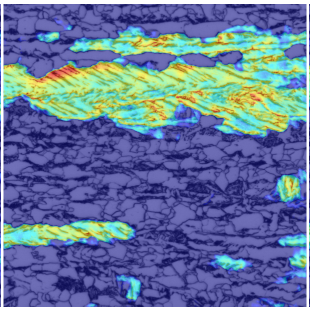

(7.20) up4.convr1

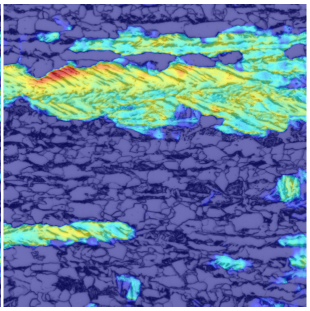

(7.21) up4.convr2

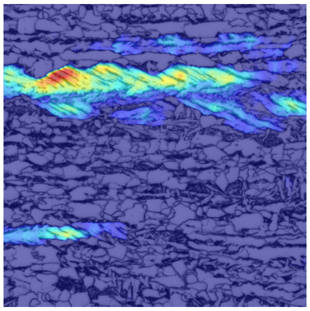

(7.22) output.seg.mask
